# Glycyl-L-histidyl-L-lysine-Cu^2+^ attenuates cigarette smoke-induced pulmonary emphysema and inflammation by reducing oxidative stress pathway

**DOI:** 10.3389/fmolb.2022.925700

**Published:** 2022-07-22

**Authors:** Qin Zhang, Liming Yan, Jingwen Lu, Xiaoming Zhou

**Affiliations:** ^1^ Department of Respiratory and Critical Care Medicine, First Hospital of China Medical University, Shenyang, China; ^2^ Department of Respiratory and Critical Care Medicine, Fourth Hospital of China Medical University, Shenyang, China; ^3^ Respiratory Department, Center for Pulmonary Vascular Diseases, Fuwai Hospital, National Center for Cardiovascular Diseases, Chinese Academy of Medical Sciences and Peking Union Medical College, Beijing, China; ^4^ Department of Respiratory and Critical Care Medicine, Shengjing Hospital of China Medical University, Shenyang, China

**Keywords:** GHK-Cu, cigarette smoke, emphysema, inflammation, oxidative stress, NF-κB, Nrf2

## Abstract

**Background:** Chronic obstructive pulmonary disease (COPD) is a common respiratory disorder manifested as chronic airway inflammation and persistent airflow limitation with the essential mechanism as inflammatory response and oxidative stress induced by toxic exposures such as cigarette smoke (CS). Glycyl-L-histidyl-L-lysine (GHK) is a nontoxic tripeptide involved in the process of healing and regeneration as a natural product. With the combination of Cu(II), glycyl-L-histidyl-L-lysine-Cu^2+^ (GHK-Cu) improves antioxidative and anti-inflammatory bioavailability, and they might offer potential therapeutic properties for COPD. Thus, the present study aimed to identify the potential effects of GHK-Cu on emphysema induced by cigarette smoke.

**Methods:** In the *in vivo* experiment, C57BL/6J mice were exposed to CS for 12 weeks to induce pulmonary emphysema. GHK-Cu was injected intraperitoneally at doses of 0.2, 2 and 20 μg/g/day in 100 µl of saline on alternative days from the 1st day after CS exposure. The effects of GHK-Cu on the morphology of CS-induced emphysema, the inflammatory response and oxidative stress were evaluated. The antioxidative effect of GHK-Cu on human alveolar epithelial A549 cells was assessed *in vitro*.

**Results:** GHK-Cu treatment attenuated the CS-induced emphysematous changes and partially reversed the matrix metalloprotein -9 (MMP-9)/tissue inhibitor of metalloproteinases-1 (TIMP-1) imbalance in the lung tissue. GHK-Cu reduced the inflammation and oxidation by decreasing the expression of inflammatory cytokines (IL-1β and TNF-α) in the bronchoalveolar lavage and the enzymatic activity of MPO and MDA in the lung homogenate while restoring the T-AOC and GSH content. Furthermore, administration of GHK-Cu reversed the increase in NF-κB expression induced by CS and increased the Nrf2 level, as an antioxidant defense component, in mice with chronic CS exposure. In CSE-exposed human alveolar epithelial A549 cells, GHK-Cu also inhibited oxidative stress by suppressing MDA levels and restoring T-AOC and GSH levels, which were modulated by upregulating Nrf2 expression.

**Conclusion:** GHK-Cu treatment attenuated CS-induced emphysema by anti-inflammation by downregulating NF-κB and antioxidation via upregulation of the Nrf2/Keap1 in lung tissues.

## Introduction

Chronic obstructive pulmonary disease (COPD) is a chronic airway disorder manifested by a persistent airflow obstruction and pulmonary parenchymal destruction, characterized by chronic airway inflammation and emphysema and finally causing respiratory failure (Adeloye et al., 2015; Wang et al., 2016a; Wang et al., 2016b). Nearly 6% of global deaths were attributed to COPD in 2012 (Li et al., 2017; X u et al., 2013; Yu et al., 2016; Wang et al., 2018). Cigarette smoke (CS) is one of the most common and important risk factors for COPD (Mannino and Buist, 2007). With the lung infiltration of neutrophils and macrophages triggered by CS, proinflammatory cytokines such as interleukin -1β(IL-1β) and tumor necrosis factor-α (TNF-α) induce a cascade of amplification effects of inflammation, and activate nuclear factor-κ B (NF-κ B) (Di Stefano et al., 2002). In addition, CS contains a large amount of oxidants (Pryor and Stone, 1993), and COPD patients have high levels of oxidative stress in their lungs (Zuo et al., 2014). The increased redox imbalance could result from oxidants in CS (Suzuki et al., 2008) and activated inflammatory cells (Boutten et al., 2010). Nuclear factor erythroid 2-related factor 2 (Nrf2) as the key transcription factor playing an essential role in redox balance and depleted in COPD patients (Deshmukh et al., 2017), modulates the expression of approximately 100 genes functioning the state of redox, inducing the transcription of glutathione (GSH) (Osburn et al., 2006).

Glycyl-L-histidyl-L-lysine (GHK) is a natural component of human plasma (Pickart and Thaler, 1973), with its level indicating the capacity of regeneration of tissue such as wound healing and skin remodeling (Pickart, 2008). GHK could form the tripeptide-copper complex glycyl-L-histidyl-L-lysine-Cu^2+^ (GHK-Cu) by the good affinity for Cu(II), which proved to exhibit good antioxidant and anti-inflammatory capabilities (Pickart, 2008). Thus, the GHK-Cu complex might gain more benefit than GHK alone since the copper supplement reduced the risk of oxidative damage (Arul et al., 2005) and effectively neutralized damaging oxygen-derived free radicals (Alberghina et al., 1992).

We have reported on the anti-inflammation and anti-fibrosis effects of the tripeptide, GHK, in pulmonary fibrosis (Zhou et al., 2017). Since abnormal lung regeneration and repair are potential processes in the pathogenesis of COPD, and the GHK-Cu complex plays a protective role in regeneration and wound healing, it is of significance to confirm whether GHK-Cu is effective in COPD as a protective agent against cigarette smoke-induced emphysematous destruction. Thus, in this study, we assessed the potential therapeutic effect of GHK-Cu against CS-induced lung inflammatory and oxidative response and emphysematous changes and explored the potential signaling pathway.

## Methods

### Experimental protocols

Sixty C57BL/6J male mice (18–20 g, 8–10 weeks, Changsheng Biotechnology Company, Liaoning, China) were housed and fed at the First Hospital of China Medical University, the Institute of Respiratory Disease, under quiet and controlled specific pathogen-free conditions. Mice were randomly divided into the following five groups: ① normal control group; ② CS group; ③ CS+0.2 μg/g GHK-Cu (G-L); ④ CS+2 μg/g GHK-Cu (G-M); and ⑤ CS+20 μg/g GHK-Cu (G-H). In the three CS + GHK-Cu groups, GHK-Cu (with purity >99.55%, China Peptides Co., Ltd. Shanghai, China) in 100 µl of saline was injected intraperitoneally (i.p.), while the mice from the normal control group and CS group were administered (i.p.) 100 µl of saline every other day. Mice were chronically exposed to filtered air for control group or to CS (20 cigarettes×twice/day, 6 days/week) from Marlboro cigarettes (Philip Morris Companies, 0.8 mg of nicotine, 10 mg of tar, and 10 mg of carbon monoxide per cigarette) for the other groups. All mice were sacrificed after 12 weeks of CS exposure or air exposure. The selected GHK-Cu doses were calculated according to the concentration in human plasma (Pickart et al., 1980) and the content reported in a previous study (Park et al., 2016).

The experimental procedures were performed complying with the Guide for the Care and Use of Laboratory Animals of China Medical University and approved by the Animal Care and Use Committee of China Medical University (Issue No. KT2021639).

### Lung tissue and sample preparation

In each group, the left lungs of 6 mice were infused with 0.5 ml PBS three times for the harvest of bronchoalveolar lavage (BAL) fluid, while the left lung tissues of the other 6 mice were inflated with 10% neutral formaldehyde at positive pressure (25 cm H_2_O) (Glynos et al., 2018; Kubo et al., 2019). After the ligation of left bronchus and the removal of the left lung tissues, the left lung tissues were immersed and fixed in 10% neutral formaldehyde for 48 h, followed by standard procedures of paraffin embedding and sections preparation. The right lung tissues were removed and used for the measurement of the antioxidative index and western blot analysis.

### Histology

The paraffin sections (4 μm) were stained with hematoxylin and eosin (H&E). Assessment of the emphysema morphology of the lungs harvested from different groups was performed based on the linear mean intercept (Lm) (×100 magnification) (Thurlbeck, 1967) and mean alveolar number (MAN) (Xu et al., 2004) (×100 magnification) as described.

### Immunohistochemistry

The expression of matrix metalloprotein -9 (MMP-9) in the lung section was identified by immunohistochemistry staining with the primary antibody of MMP-9 (1:1,000 dilution, Abcam), according to the manufacturer’s instructions.

### Inflammatory cytokine analyses in BAL fluid

To obtain BAL fluid, the trachea was exposed, followed by the ligation of the right main bronchus. A 23-G needle was used to slowly inject 0.5 ml cold PBS with 0.1 mM EDTA into the left lung, and then the BAL fluid was collected from the lungs. IL-1β and TNF-α assessment in the supernatant of BAL fluid samples was determined using commercially available ELISA kits (CUSABIO, Wuhan, China).

### Preparation of cigarette smoke extract

CSE was prepared according to the previous study (Chen et al., 2009). One nonfiltered Marlboro cigarette was burned, and then the smoke was passed through 4 ml PBS. The extract was filtered using a filter with 0.22-μM pores and the pH was adjusted between 7.00 and 7.40 after filtering it. Fresh CSE was prepared before each injection.

### *In vitro* experiment

Human alveolar epithelial A549 cells were purchased from the China Infrastructure of Cell Line Resource. A549 cells were cultured in RPMI 1640 (10% FBS, 2 mM L-glutamine, 100 U/ml penicillin, and 100 μg/ml streptomycin) at 37°C in an atmosphere of 5% CO_2_. A549 cells were treated with 5 or 10 μM GHK-Cu and then stimulated with 5% CSE for up to 24 h for further experiments.

### The measurement of antioxidant index and myeloperoxidase activity in the lung homogenate

Biochemical assays were carried out in the supernatants of homogenates of lung tissues and A549 cell samples. The supernatants were collected from sample homogenates following centrifugation at 2,500 rpm and 4°C for 10 min. The supernatant was used to detect antioxidant biomarkers, such as the glutathione (GSH), total antioxidant capacity (T-AOC), content and malondialdehyde (MDA) levels, according to the manufacturer’s instructions (Nanjing Jiancheng Bioengineering Institute, Nanjing, China). Bicinchoninic acid (BCA) protein assay kit (Beyotime Institute of Biotechnology) was used to determine the protein concentration, with bovine serum albumin as the standard. In addition, MPO activity was estimated using an MPO Detection Kit (Nanjing Jiancheng Bioengineering Institute, China).

### Western blot analysis

Proteins were extracted from the stored lung tissue samples and A549 cell samples using radioimmunoprecipitation assay (RIPA) lysis buffer (Cell Signaling, US). To determine the expression of translocated Nrf2 and NF-κB in the nucleus, and the levels of Nrf2, Keap1, heme oxygenase-1 (HO-1) and p-IκBα in the cytoplasm, nuclear and cytoplasmic extracts from the lung tissue were prepared with a previously described method (Rajendrasozhan et al., 2010). All of the western blot data represent triplicate experiments. Densitometric analysis was performed using ImageJ software, version 6.0 (National Institutes of Health, Bethesda, MD, United States).

### Statistical analysis

The results are depicted as the mean ± SEM. One-way analysis of variance (ANOVA) followed by the Newman-Keuls comparison test using GraphPad Prism software (CA, US) was employed for analysis. *p* < 0.05 was considered significant.

## Results

### GHK-Cu administration alleviated CS-induced emphysematous changes

**FIGURE 1:**
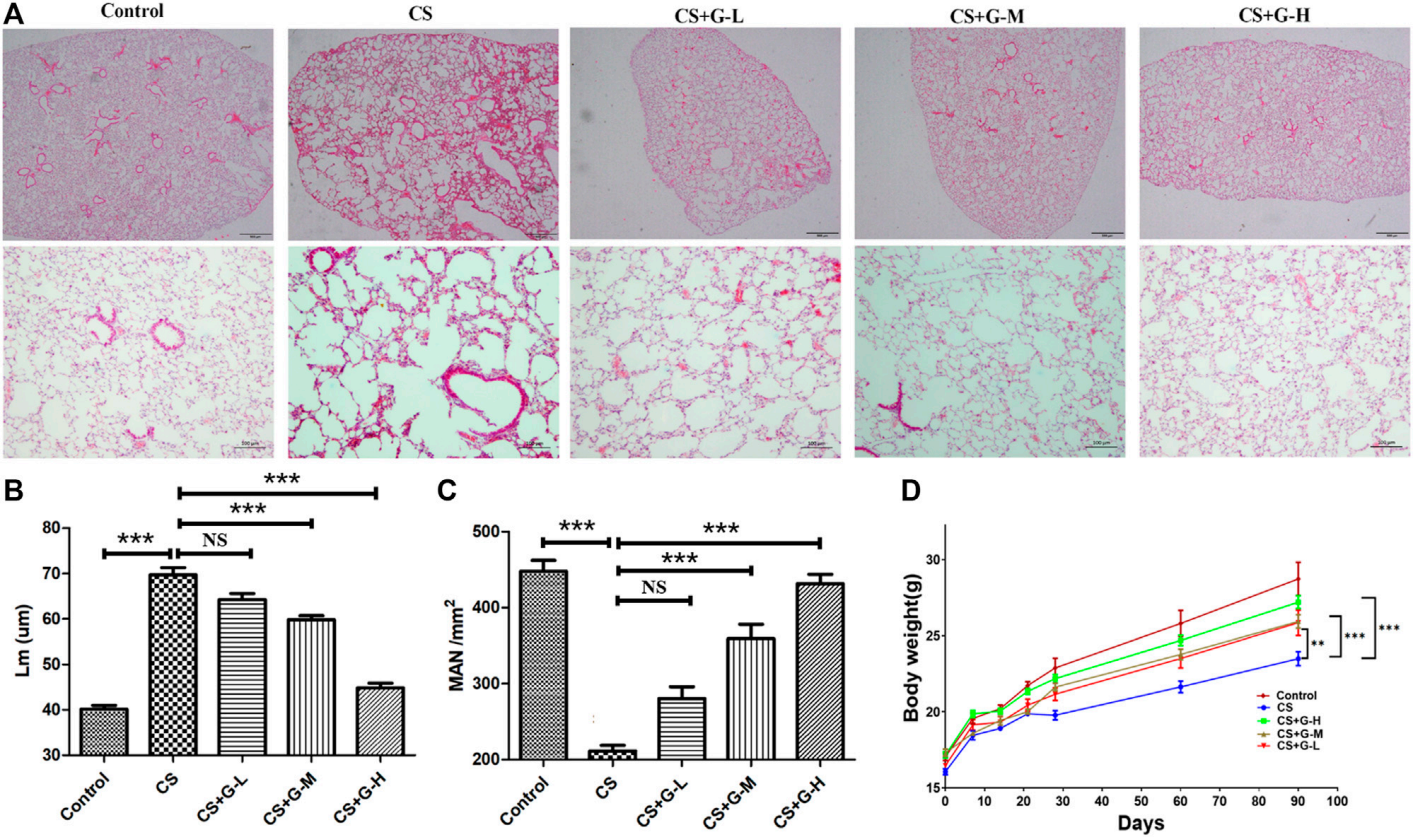
GHK-Cu alleviates cigarette smoke-induced emphysematous changes. **(A)** Photomicrograph of HE-stained slides for histological analysis of mice after chronic exposure to cigarette smoke or room air. Representative images of micrographs at ×20 and ×100 magnification. **(B,C)** Histological assessment of the sections was determined using Lm **(B)** and MAN **(C)** (*n* = 6 mice/group). **(D)** The body weight was measured (*n* = 10–12 mice/group). **p < 0.01, ***p < 0.001, and NS means not significant (*p* > 0.05).

We assessed the efficacy of GHK-Cu administration in CS-induced emphysema. CS exposure significantly extended Lm when compared to control group. Treatment with medium and high dosages of GHK-Cu significantly reduced the extension of Lm compared to the increases in the mice in the CS group, while it increased the MAN compared to the reduction of MAN in the CS group (Figures 1A–C), indicating that GHK-Cu reduced the morphological changes due to emphysema caused by CS exposure. GHK-Cu also partially reversed the CS-induced body weight decrease (Figure 1D).

### GHK-Cu restored the MMP-9/TIMP-1 balance

**FIGURE 2:**
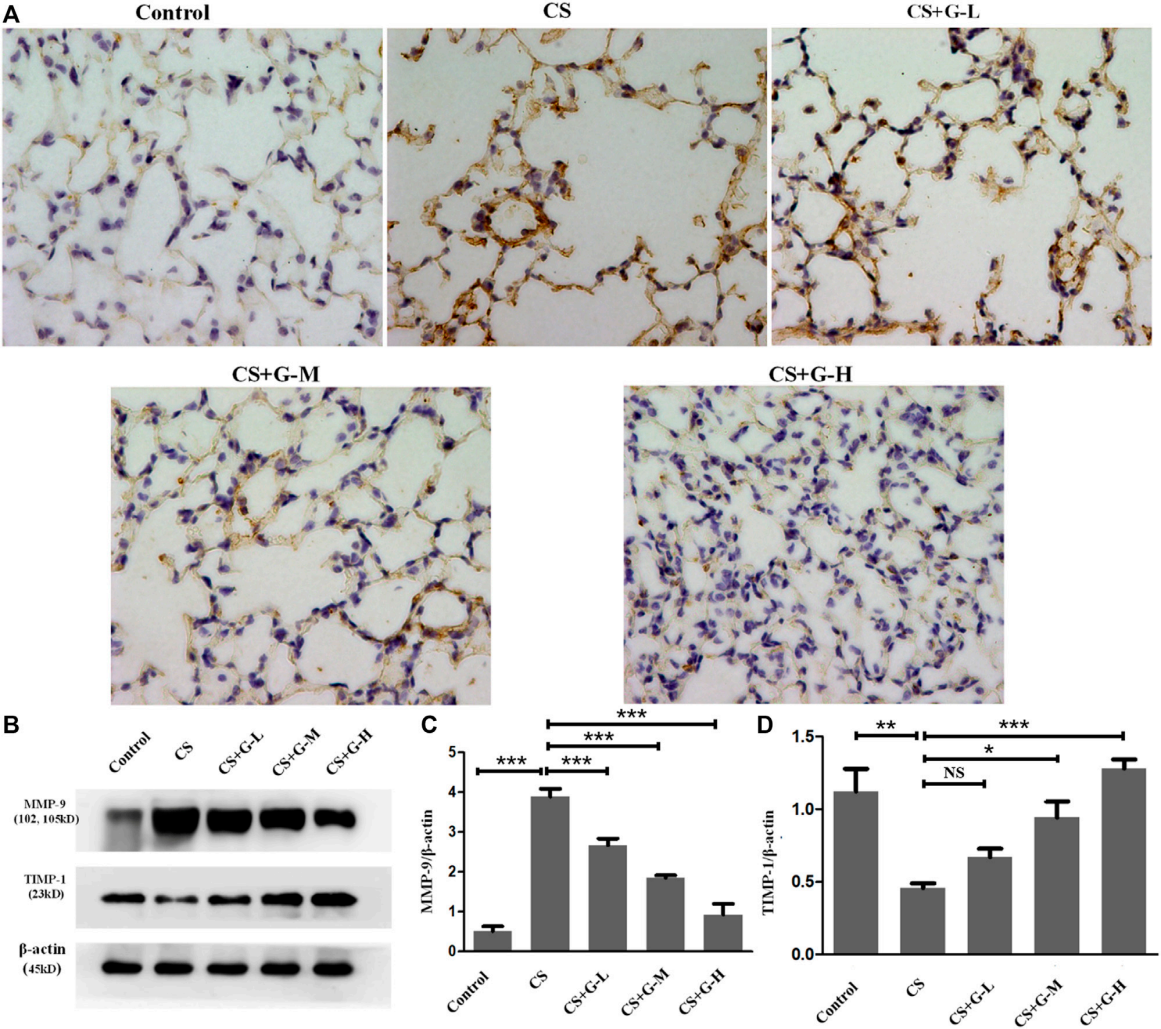
GHK-Cu restored the MMP-9/TIMP-1 balance in mice with cigarette smoke exposure. **(A)** Photomicrograph of immunohistochemical stained slides for MMP-9 from mice with chronic cigarette smoke or room air exposure. Representative images of micrographs at ×400 magnification. **(B)** Expression of MMP-9 and TIMP-1 in lung tissue from different groups was assessed by western blot. **(C,D)** Western blotting analysis of MMP-9 **(C)** and TIMP-1 **(D)** protein expression in the lung tissue of mice in different groups. *p < 0.05, **p < 0.01, ***p < 0.001, and NS means not significant (*p* > 0.05).

Emphysema is reported to be consequence of destroyed lung elastin and degradation of collagen matrix, presenting as the imbalance of MMPs and tissue inhibitors of MMP (TIMPs) (Houghton, 2015). Thus, MMPs and TIMPs are considered a hallmark of COPD pathogenesis (Cornwell et al., 2010). By immunohistochemical staining and western blot analysis, an increase in MMP-9 expression in the CS group was observed compared to that in the control group, and pretreatment with GHK-Cu reduced MMP-9 expression compared to that in the CS group (Figures 2A–C). The protein expression of TIMP-1 was decreased in the CS group when compared with the control group, whereas pretreatment with medium and high dosages of GHK-Cu increased it when compared to the CS group (Figures 2B,D).

### Treatment with GHK-Cu ameliorated CS-induced lung inflammation

**FIGURE 3:**
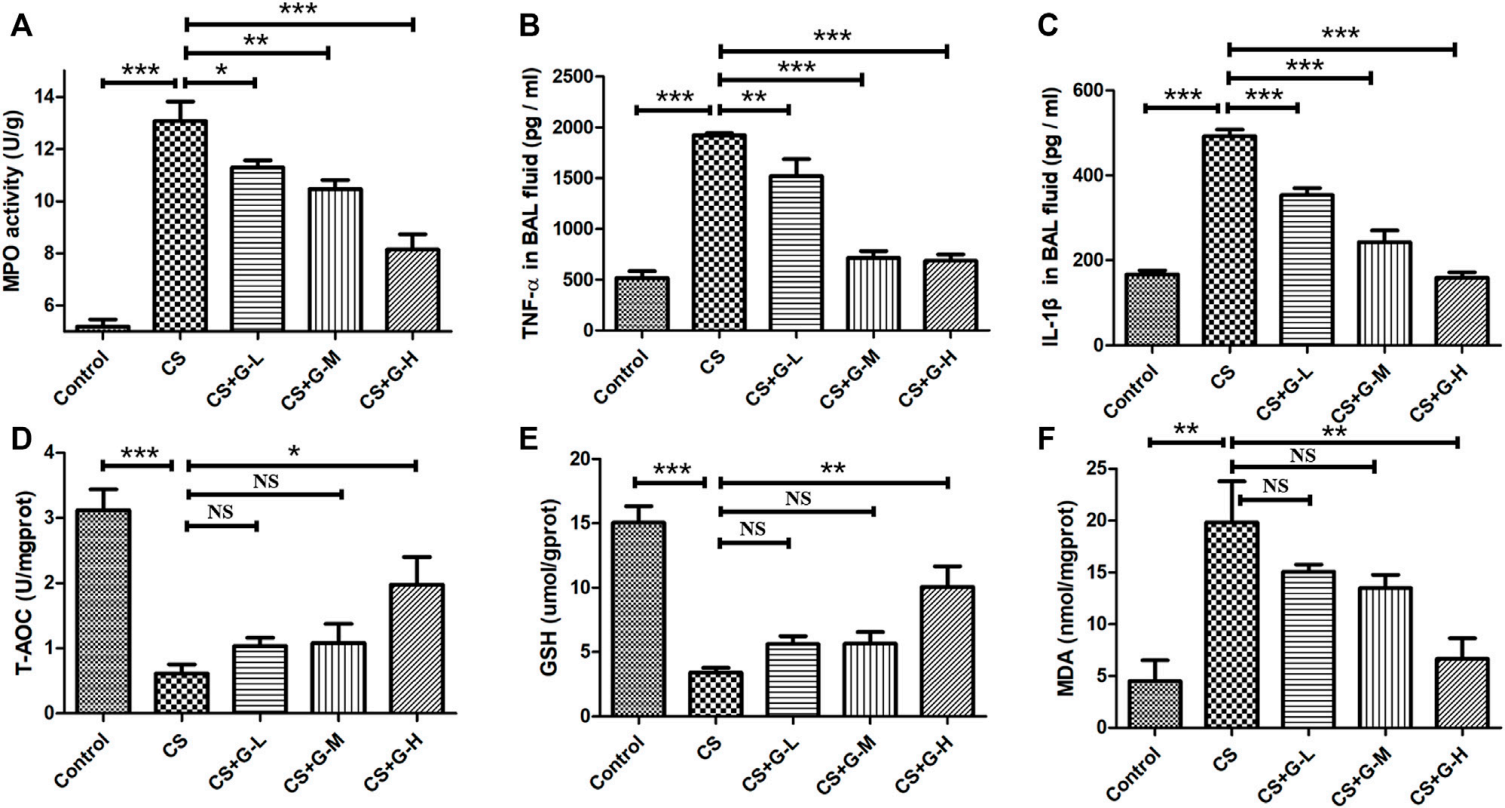
Treatment with GHK-Cu ameliorated lung inflammation and oxidative stress induced by cigarette smoke. **(A)** MPO activity in lung tissue was assessed following chronic exposure to cigarette smoke or room air. **(B,C)** Proinflammatory cytokines, including TNF-α **(B)** and IL-1β **(C)**, in BAL fluid supernatant were measured by ELISA (*n* = 6 mice/group). **(D–F)** The levels of T-AOC **(D)**, GSH **(E)** and MDA **(F)** in lung homogenate were detected in mice of different groups (*n* = 3 mice/group). *p < 0.05, **p < 0.01, ***p < 0.001, and NS means not significant (*p* > 0.05).

Neutrophilic inflammation was assessed through the activity of MPO (Ndrepepa, 2019). Thus, we explored the MPO activity in lung tissue with the treatment of GHK-Cu under the exposure of CS and found that GHK-Cu administration reduced the increase in MPO enzymatic activities induced by CS exposure (Figure 3A).

In addition, we determined the proinflammatory cytokines associated with COPD in BAL fluid. No difference was found in the amount of BAL fluid among the groups. CS exposure was shown to lead to the increase of IL-1β and TNF-α in BAL fluid, while GHK-Cu reduced the release of these inflammatory cytokines in BAL fluid (Figures 3B,C).

### CS-induced oxidative stress in mice with emphysema was prevented partially by the complex of GHK-Cu

Oxidative stress is one of the essential factors in emphysema changes (Han et al., 2011), so we evaluated the oxidative stress levels in CS-induced emphysema. T-AOC, MDA and GSH, biomarkers of oxidative stress, were evaluated by detecting the levels in the lung homogenate. The levels of T-AOC, GSH and MDA are shown in Figures 3D–F. CS challenge stimulated the reduction of T-AOC and GSH in lung tissue, while high dosage of GHK-Cu pretreatment relieved the decrease of these expression levels in lung tissue. Conversely, CS increased the activity of MDA in the lung tissue of mice in the CS group compared to the control group, while GHK-Cu pretreatment as in the high dosage inhibited the augment of MDA activity induced by CS.

### GHK-Cu upregulated the Nrf2/Keap1 pathway

**FIGURE 4:**
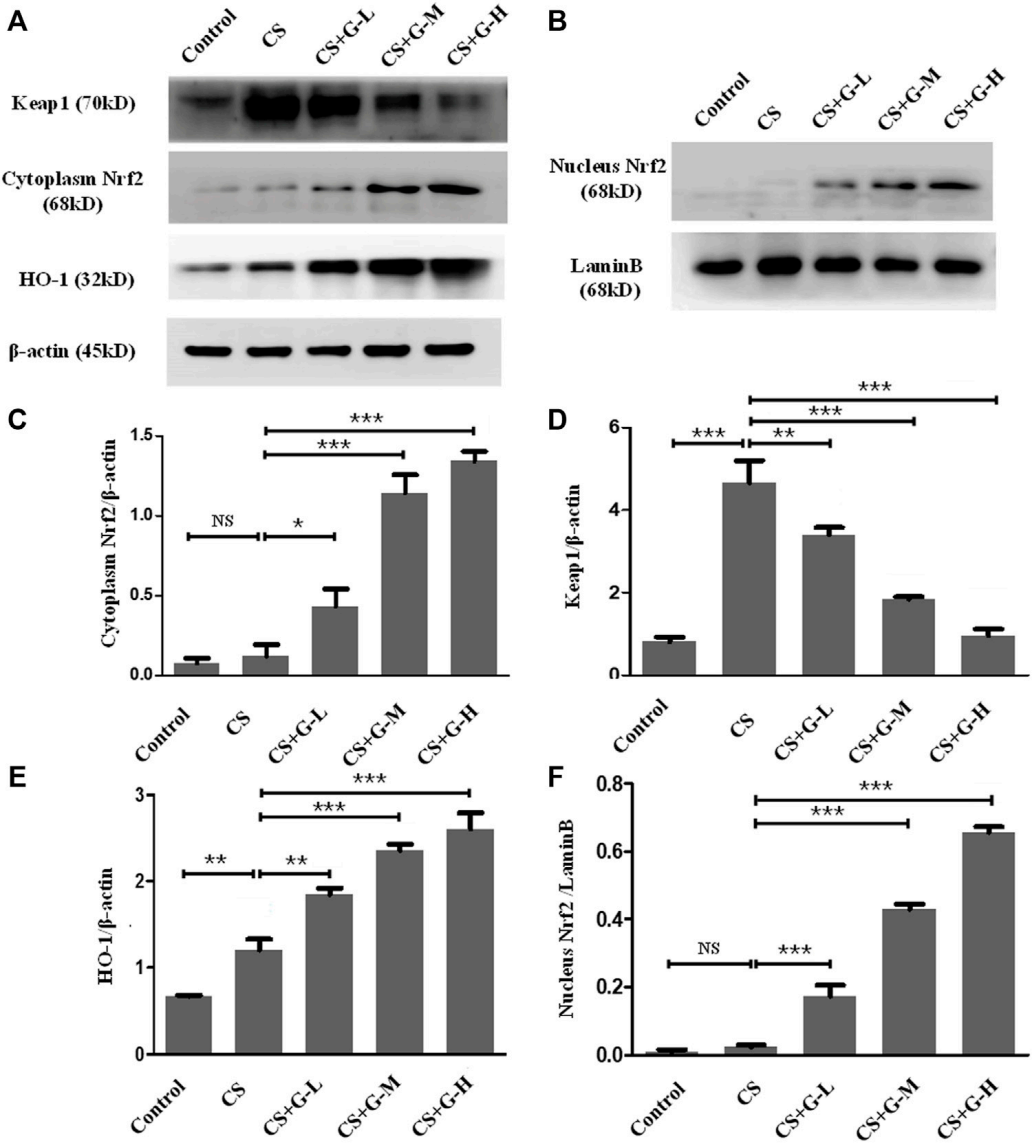
GHK-Cu upregulated the Nrf2/Keap1 pathway in mice with chronic cigarette smoke exposure. **(A,B)** The protein expression of Nrf2/Keap1 pathway in the cytoplasm and nuclei of lung tissue in mice from different groups was evaluated by western blotting. **(C–E)** Western blotting analysis of Nrf2 **(C)**, Keap1 **(D)** and HO-1 **(E)** expression in the cytoplasm of the lung tissue from mice in different groups. **(F)** Western blotting analysis of Nrf2 in the nuclei of lung tissue cells in mice from different groups was assessed. *p < 0.05, **p < 0.01 and ***p < 0.001, and NS means not significant (*p* > 0.05).

Nrf2 is attached to Keap1 in the cytoplasm under normal circumstances, while in the condition of stress, the stimuli of oxidative stress activate the separation of Nrf2 and Keap1 and translocate Nrf2 to the nucleus and switch on transcriptional activities (Itoh et al., 1997). To evaluate this pathway in the emphysema model, the expression of Nrf2 and Keap1 protein was measured by western blotting in the cytoplasm and nucleus, respectively. Pretreatment with GHK-Cu strongly promoted Nrf2 expression while significantly reducing the level of Keap1 in the cytoplasm compared to the CS group (Figures 4A,C,D). Next, the level of Nrf2 in the nucleus was measured, and our results revealed that GHK-Cu markedly increased its expression (Figures 4B,F). As HO-1 is produced by the transcription of Nrf2, effecting as an antioxidant enzyme (Itoh et al., 1997), we also measured the level of HO-1. As expected, GHK-Cu administration induced a significant augment in the expression of HO-1 (Figure 4E).

### GHK-Cu prevented the activation of NF-κB and i-NOS

**FIGURE 5:**
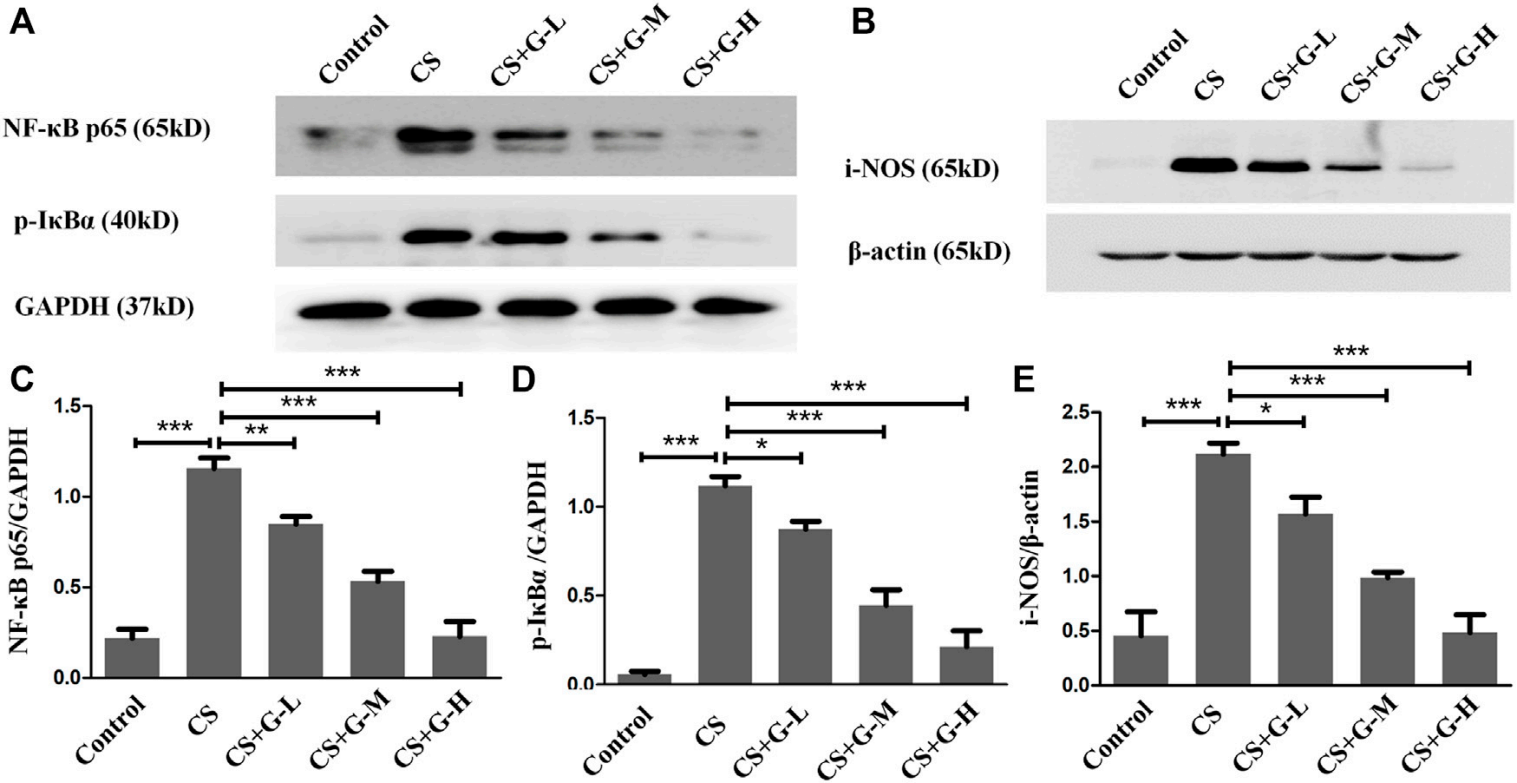
GHK-Cu prevented the activation of NF-κB and i-NOS induced by cigarette smoke. **(A,B)** The expression of NF-κB p65, p-IκBα and i-NOS in lung tissue in mice from different groups was assessed by western blotting. **(C–E)** Western blotting analysis of NF-κB p65 **(C)**, p-IκBα **(D)** and i-NOS **(E)** expression in the lung tissue of mice from different groups. *p < 0.05, **p < 0.01 and ***p < 0.001.

The action influence of GHK-Cu on CS-induced NF-κB activation was assessed by the level of phosphorylation of IκBα and NF-κB p65. The results indicated that CS exposure notably amplified NF-κB p65 expression, and a notable reduction in NF-κB p65 expression was observed in the CS + GHK-Cu groups compared with the CS group. In addition, we examined the level of phosphorylation of IKBα and found that GHK-Cu downregulated the levels of NF-κB P65 with decreasing phosphorylation of its inhibitor IκBα in the lungs (Figures 5A,C,D). It was reported that NF-κB-regulated i-NOS activation is associated with inflammation (Vaporidi et al., 2010; Tabassum et al., 2015). In the present study, pretreatment with GHK-Cu could decrease the i-NOS level via the NF-κB pathway (Figures 5B,E).

### *In vitro* inhibition of oxidative stress in human alveolar epithelial cell by GHK-Cu via the upregulation of Nrf2 level

**FIGURE 6:**
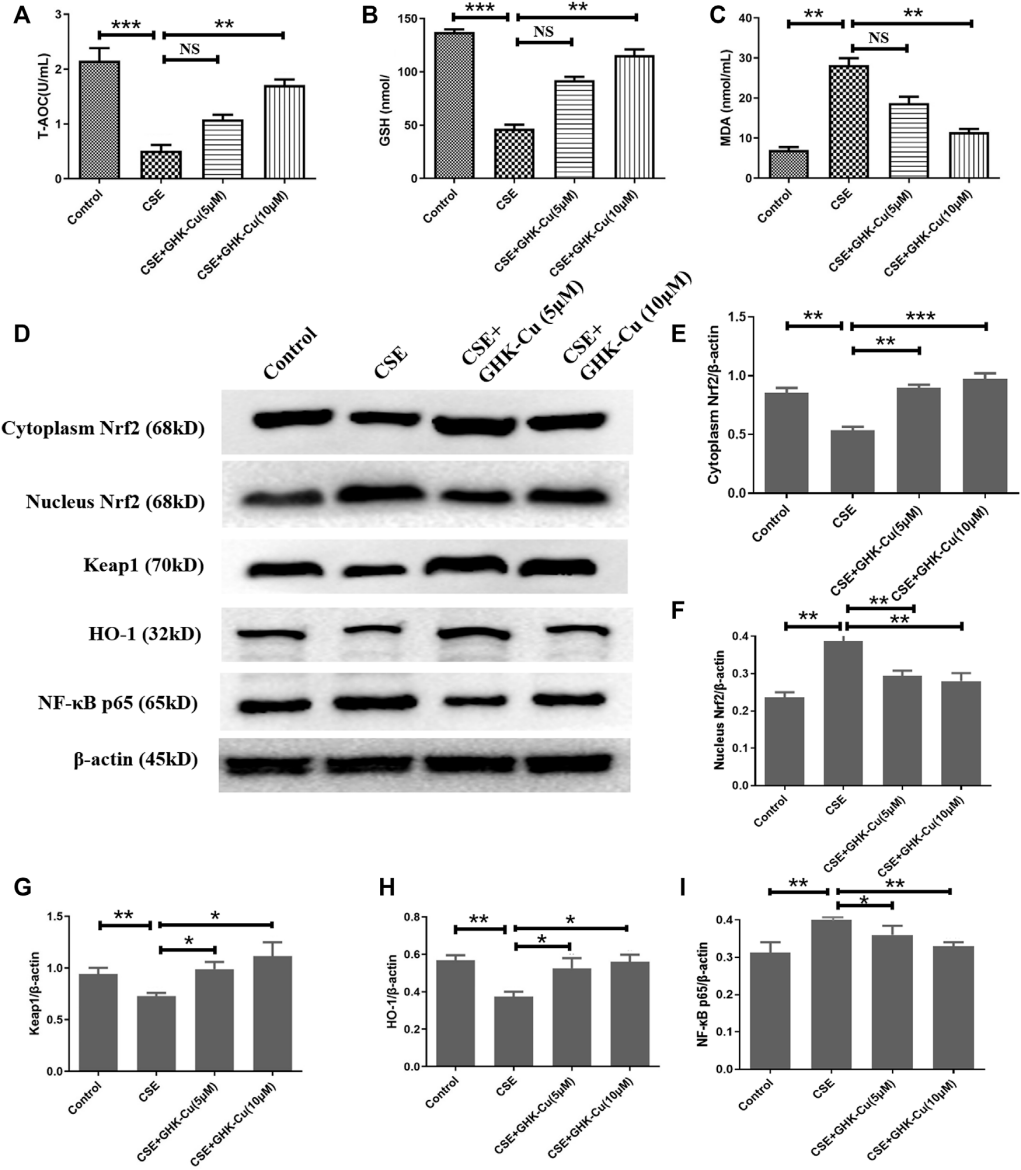
GHK-Cu inhibits oxidative stress in human alveolar epithelial cells by upregulating Nrf2 levels *in vitro*. **(A–C)** The levels of T-AOC **(A)**, GSH **(B)** and MDA **(C)** in A549 cells were detected. **(D)** The protein expression of Nrf2/Keap1 pathway and NF-κB p65 in the cytoplasm and nuclei of A549 cells were measured. **(E–G)** Western blotting analysis of cytoplasmic Nrf2 **(E)**, nuclear Nrf2 **(F)**, Keap1 **(G)**, HO-1 **(H)** and NF-κB p65 **(I)** protein expression in the cytoplasm of the lung tissue from mice in different groups. *p < 0.05, **p < 0.01 and ***p < 0.001, and NS means not significant (*p* > 0.05).

After applying 5% CSE into A549 cells for 24 h, we found that the MDA level was increased, and the levels of T-AOC and GSH were decreased compared with those in A549 cells without CSE exposure, indicating that CSE stimulates an increase in oxidative stress in human alveolar epithelial cells. GHK-Cu alleviated the oxidative stress level in A549 cells in a concentration-dependent manner (Figures 6A–C).

CSE stimulation depleted Nrf2 in A549 cells. Consistent with the results observed in *in vivo* experiments, GHK-Cu significantly restored Nrf2 expression in the cytoplasm and reduced it in the nucleus while significantly reducing Keap1 levels in the cytoplasm compared with the CSE-exposed cells without GHK-Cu (Figures 6D–F). Additionally, GHK-Cu treatment notably upregulated HO-1 expression (Figure 6G) and downregulated NF-κB p65 expression (Figure 6I).

## Discussion

COPD is a common disease usually related to significant exposure to noxious particles or gases (Halpin et al., 2021). Emphysema is a pathologically morphological abnormality that is often used to describe the structural changes seen in COPD patients. To date, the mechanism of COPD is not completely understood. Inflammation, oxidation, together with the imbalance of MMPs/TIMPs, has been reported widely to involve in the pathogenesis process associated with COPD. To the best of our knowledge, animal models have been mostly built by exposure to CS, intranasal instillation of elastase and intraperitoneal injection of CSE (Barreiro et al., 2019). Due to its great stability and reproducibility, we chose CS exposure as a model of emphysema. In our study, chronic CS exposure led to marked airspace enlargement and administration of GHK-Cu provided visible protection against emphysema. As far as we know, this report is the first to stress the role of GHK-Cu in an animal model of CS-induced emphysema. In addition, our data suggested that GHK-Cu provides potential protection against oxidative stress and inflammation induced by CS exposure.

Airway inflammation takes an essential part in the pathogenesis of COPD. After chronic CS exposure, a cascade of inflammatory processes is triggered. The inflammatory cellular infiltration, such as neutrophils and macrophages, mediating prolonged airway inflammation can be induced, and inflammatory cytokines are released, such as TNF-α as an initial proinflammatory cytokine stimulating the expression of downstream cytokines, such as IL-1β(6). Our study indicated that medium and high dosages of GHK-Cu markedly suppressed the secretion of TNF-α and IL-1β. MPO is sufficiently expressed in neutrophils; and as a microbicidal peroxidase, it can induce the strong response of oxidation at the site of inflammation by catalyzing hypochlorous acid (Zhang et al., 2002). Previous studies have demonstrated that neutrophilic inflammation and MPO activity in the lung and sputum of COPD patients correlate well with disease progression (Park et al., 2013; Zhu et al., 2014). Consistent with this result, our result indicated the MPO activity of lung tissue markedly rose upon CS exposure. We discovered that medium and high dosages of GHK-Cu significantly decreased MPO activity, consistent with the proinflammatory factors secreted in BAL fluid. We verified that GHK-Cu had an important anti-inflammatory effect in CS-induced emphysema in mice.

NF-κB regulates many genes in the inflammatory response that can be promoted by proinflammatory stimuli, such as CS and TNF-α (Rhee et al., 2007). Most of the time, the primary form of NF-κB localizes in the cytoplasm and is inactive when binding to its inhibitory protein known as IκBα (Li et al., 2012). When stimuli activate NF-κB, IκBα can be degraded. The NF-κB released and translocated into the nucleus exerts its pro-inflammation function. The activation of NF-κB during inflammation increases the levels of proinflammatory factors and other proinflammatory genes (Lawrence et al., 2001). Evidence has suggested that NF-κB regulates i-NOS, which is closely associated with inflammation. Furthermore, among several transcription factors related to i-NOS expression, NF-κB is considered to be one of the most essential factors (Kinaci et al., 2012; Tabassum et al., 2015). Measurement of the NF-κB activation in our *in vivo* experiment showed that GHK-Cu blocked the transcriptional activity of NF-κB through suppression of NF-κB by altering the phosphorylation of its inhibitor i-κBα. In addition, pretreatment with GHK-Cu was able to reduce i-NOS expression, consistent with the repression of NF-κB pathway activation.

CS exposure causes both the release of proinflammatory cytokines and a large number of reactive oxygen species (ROS), leading to the damage of both oxidative and aggravating inflammation, thus resulting in the morphological changes in emphysema after long-term exposure. Current pharmacotherapies for COPD, such as bronchodilators, can only relieve symptoms, and they fail to prevent disease progression effectively. Therefore, the potential mechanisms of oxidative response help to find the novel and more effective therapies for CS-induced emphysema. Persistent inflammation and cigarette smoke are primary sources of ROS in the lung, causing redox imbalance and peroxidation damage to lipids, proteins, and nucleic acids (Kirkham and Barnes, 2013). As an important pulmonary antioxidant, GSH is important for maintaining epithelial integrity, and its deficiency results in airway and alveolar damage (Espinosa-Diez et al., 2015). We supposed that Nrf2-mediated biosynthesis of GSH is impaired by the stimulation of CS, whereas GHK-Cu modulates Nrf2, attenuates pulmonary oxidative stress and thereby restores GSH depleted by CS. In our study, we verified this hypothesis by investigating the protective role of GHK-Cu in mouse lung tissue and in human alveolar epithelial cell *in vitro*. MDA, is widely used as a marker of oxidative stress due to its role as the fatty acid peroxidation product. Previous reports have revealed that an increase of MDA level was observed in the sputum and serum of COPD patients and correlated well with disease progression (Montaño et al., 2010; Žuža et al., 2022). GHK was shown to completely block Cu(II)-dependent oxidation of low density lipoproteins (LDL) (Pickart and Margolina, 2018). Our results demonstrated that CS exposure led to an enhancement of oxidative stress reflected by increased MDA level, associated with diminished expression of Nrf2, GSH and T-AOC activity in the lung tissues; while CSE exposure led to diminished expression of Nrf2 in human alveolar epithelial cell.

Nrf2 is a key regulator of redox homeostasis in the gastrointestinal tract, brain, kidney, liver, and skin, etc. And it regulated up to 500 genes, which targeted at the function of proteins for maintenance of redox balance, detoxifying enzymes, and metabolic enzymes (Pall and Levine, 2015; Furfaro et al., 2016). Upon the upregulation and activation of Nrf2, the heterodimers of nuclear Nrf2 and small Maf proteins form to recognize antioxidant response elements (AREs), and then recruit key factors for transcription of Nrf2 target gene including HO-1 (Hayes et al., 2010). This process explains why the main function of Nrf2 is to prevent oxidative stress by inducing the production of antioxidants and why HO-1 determinedly is regulated by Nrf2 activation (Joo Choi et al., 2014). In this study, exposure to cigarette smoke remarkably dampened Nrf2 nuclear translocation in the lungs, and GHK-Cu strongly augmented nuclear Nrf2 levels to greater than those of the control mice. Consistently, cigarette smoke downregulated HO-1 expression in the CS group mice, and GHK-Cu markedly increased the expression of HO-1. In addition, in human alveolar epithelial cell, GHK-Cu significantly enhanced the expression of HO-1 which has been reduced by the stimulation of CSE. Therefore, it was concluded that GHK-Cu might be a therapeutic candidate for COPD with the role of anti-inflammation and anti-oxidation.

The Keap1-Nrf2 pathway is the principal protective response to oxidative and electrophilic stresses. Nrf2 regulation is achieved through various pathways, including Keap1-dependent and Keap1-independent pathways (Audousset et al., 2021). The latter regulates the Nrf2-ARE pathway mainly through phosphorylation sites and is considered a critical regulatory factor of Nrf2 nuclear accumulation, nuclear rejection, and degradation. However, the Keap1-dependent regulation of Nrf2 activity is a sophisticated dual negative control mechanism. Under homeostatic conditions, Keap1 forms part of an E3 ubiquitin ligase, which tightly regulates the activity of the transcription factor Nrf2 by targeting it for ubiquitination and proteasome-dependent degradation. In response to stress, an intricate molecular mechanism facilitated by sensor cysteines within Keap1 allows Nrf2 to escape ubiquitination, accumulate within the cell, and translocate to the nucleus, where it can promote its antioxidant transcription program. However, overexpression of Keap1 was shown to repress the nuclear accumulation and transcriptional activity of Nrf2, while the addition of phase II inducers, which could upregulate Nrf2, was able to relieve this repression. One study indicated that Nrf2 knockout may reduce Nrf2 entry into the nucleus and fail to initiate the transcription of downstream molecules, thereby exacerbating inflammation and autophagy in a severe pancreatitis induced acute lung injury model; however, the expression of Keap1 was higher in Nrf2^−/−^ mice than in WT mice (Kong et al., 2021). In contrast, the expression of NQO1 and Ho-1 was downregulated after Nrf2 knockout. This result is consistent with our results of *in vivo* experiments indicating the negative control mechanism of the Keap1-Nrf2 axis. The reason for the contradictory results in the *in vitro* experiments was probably the difference in the severity of the oxidation stress. Under moderate oxidative stress, Keap1 is inhibited, allowing Nrf2 to be translocated to the nucleus, where it acts as an antioxidant. However, under unusually severe oxidative stress, the Keap1-Nrf2 mechanism becomes disrupted and results in cell and tissue damage. These paradoxical results could be observed in some other studies, such as the Keap1-Nrf2 axis in acute lung injury, in which Keap1 expression increased in some animal and *in vitro* experiments (Huang et al., 2020; Li et al., 2021), while Keap1 expression decreased in some animal and *in vitro* experiments (Yan et al., 2019; Zheng et al., 2021; Zhu et al., 2021).

MMPs and TIMPs imbalance plays a key role in the destruction of lung parenchyma (Mocchegiani et al., 2011). When the upregulating MMP-9 cannot be offset by TIMP-1 abundantly, the degradation of elastin and other extracellular matrix components occurs in the alveolar walls and finally leading to emphysema (Pardo and Selman, 1999; Muroski et al., 2008). As our expectations, our result showed MMP-9 had a significant increase in the CS group. In contrast, the expression of MMP-9 in mouse lungs was suppressed by GHK-Cu which was increased in CS group. In parallel, the increase in TIMP-1 in the CS + GHK-Cu groups, together with the reduction in MMP-9, indicated low levels of extracellular matrix protein degradation, reflecting less tissue damage.

## Conclusion

Our results provide evidence that GHK-Cu pretreatment is protective against CS-induced emphysema due to its anti-inflammatory and antioxidative effects. In addition, our data provide evidence that the protective effects are likely related to modulation of the NF-κB and Nrf2/Keap1 pathways. Consistent with the results observed in *in vivo* experiments, GHK-Cu significantly inhibited oxidative stress in human alveolar epithelial cell *in vitro*.

## Data Availability

The original contributions presented in the study are included in the article/supplementary material, further inquiries can be directed to the corresponding authors.

## Abbreviations

COPD, Chronic obstructive pulmonary disease; GHK, Glycyl-L-histidyl-L-lysine; CS, Cigarette smoke; MMP, matrix metalloprotein; TIMP-1, tissue inhibitors of MMP-1; IL, interleukin; NF-κ B, nuclear factor-κ B; TNF-α, tumor necrosis factor-α; Nrf2, Nuclear factor erythroid 2-related factor 2; GSH, glutathione; BAL, bronchoalveolar lavage; Lm, linear mean intercept; MAN, mean alveolar number; CSE, cigarette smoke extract; T-AOC, total antioxidant capacity; MPO, myeloperoxidase; BCA, bicinchoninic acid; MDA, malondialdehyde; HO-1, heme oxygenase-1; ROS, reactive oxygen species; i-NOS, inducible nitric oxide synthase.
